# Supplementary material for: Translatory and rotatory motion of exchange-bias capped Janus particles controlled by dynamic magnetic field landscapes
Source: Sci Rep. 2021 Nov 8;11:21794. doi: 10.1038/s41598-021-01351-x (PMC8575999; doi:10.1038/s41598-021-01351-x)
Supplement: Supplementary file 1 — Supplementary Information. [file 41598_2021_1351_MOESM1_ESM.pdf]

Translatory and rotatory motion of Exchange-Bias capped Janus particles controlled by dynamic magnetic field landscapes

Rico Huhnstock[1),2)], *Meike Reginka*[1),2)], *Andreea Tomita*[1)], *Maximilian Merkel*[1),2)], *Kristina Dingel*[2),3)], *Dennis Holzinger*[1)], *Bernhard Sick*[2),3)], *Michael Vogel*[1),2)], *Arno Ehresmann*[1),2)]

1) Institute of Physics and Centre for Interdisciplinary Nanostructure Science and Technology (CINSaT), University of Kassel, Heinrich-Plett-Strasse 40, D-34132 Kassel, Germany

   E-Mail: rico.huhnstock@physik.uni-kassel.de, Phone: +49 561 804-4198

2) Artificial Intelligence Methods for Experiment Design (AIM-ED), Joint Lab of Helmholtzzentrum für Materialien und Energie, Berlin (HZB) and Kassel University, Germany
3) Intelligent Embedded Systems, University of Kassel, Wilhelmshöher Allee 71-73, D-34121 Kassel, Germany

Supplementary Information

S1: Calculation of steady-state distances

For the calculation of steady-state distances between Janus particles and the surface of the underlying substrate, three relevant forces are balanced against each other, depending on whether they are attractive or repulsive forces. We take the following forces into account: the

magnetic force $\vec{F}_M(z)$ (see Eq. (2) in the manuscript) between the magnetic substrate and the magnetic cap of the particle, the van der Waals force $\vec{F}_{vdW}(z)$ originating from interactions between dipoles and/or induced dipoles and the electrostatic force $\vec{F}_{el}(z)$. Employing the Hamaker constant A_{132} for a particle of material 1 above a substrate of material 2 surrounded by a medium of material 3, the van der Waals force can be expressed as follows:

$$\vec{F}_{vdW}(z) = -\frac{A_{132} \cdot R_P}{6z^2} \cdot \left[\frac{1}{1 + 14\frac{z}{\lambda_{ret}}}\right] \cdot e_z, \qquad (1)$$

With λ_{ret} representing the retardation wavelength of the interaction, which in this case is assumed to be 100 nm. Considering the immersion of the particles and the substrate surface in an ionic liquid like water, a double-layer of opposite charges is forming at each surface, leading to an electrostatic force between the surfaces when their respective double-layers start to overlap. This force is governed by the surface potentials of the particle Ψ_P and the substrate Ψ_S, respectively, and the inverse double-layer thickness κ, given by the Debye-Hückel theory. Now, the electrostatic force can be written as:

$$\vec{F}_{el}(z) = \frac{2 \cdot \pi \cdot \varepsilon \cdot \kappa \cdot R_P}{1 - e^{-2\kappa z}} \cdot [2 \cdot \Psi_S \cdot \Psi_P \cdot e^{-\kappa z} \mp (\Psi_S^2 + \Psi_P^2) \cdot e^{-2\kappa z}], \qquad (2)$$

with ε being the permittivity of the medium. Depending on the used sign in Eq. (2), it can be differentiated between a model for the electrostatic force, where the surface potentials of both particle and substrate are assumed to be constant (upper sign) and where the surface charge density of both components is assumed to be constant (lower sign). Since both of these models describe extreme cases for the behavior of the here studied system, calculations for the electrostatic force were conducted using both approaches. In order to obtain the steady-state distance between substrate surface and Janus particle, $\vec{F}_M(z), \vec{F}_{vdW}(z)$ and $\vec{F}_{el}(z)$ were computed in dependence of z for particles with a diameter of 3 µm. For the calculation of $\vec{F}_M(z)$, the simulated magnetic stray field landscape $\vec{H}_{MFL}(x, z)$ above the substrate (retrieved from

micromagnetic simulations for the used domain configuration) was considered at the position x above the center of a domain wall. For $\vec{F}_{vdW}(z)$, a Hamaker constant of $A_{132} = 3.4 \cdot 10^{-21}$ was chosen, leading to an attractive van der Waals interaction between particle and substrate. However, in the case of $\vec{F}_{el}(z)$ a repulsive force is present, since for Ψ_S and Ψ_P the zeta potentials of PMMA (-35 mV) and glass (-35 mV) at pH = 7 were used, respectively. Balancing the sum of $\vec{F}_M(z)$ and $\vec{F}_{vdW}(z)$ in dependence on the distance between particle surface and resist surface and the repulsive force $\vec{F}_{el}(z)$ yields the stead-state distance. It is important to note, that the gravitational force F_G and the buoyancy force F_B were not considered for the calculation of the steady-state distance, since they are two to three orders of magnitude smaller for Janus particles with d = 3 μm than the discussed forces which are in the range of 10^{-10} N and 10^{-12} N for the relevant ranges of z. When finding that both models for the electrostatic force yield two clear intersections with the attractive forces curve, we take the average of both as the estimated steady-state distance.

S2: Video of magnetic Janus particle motion

Exemplary video demonstrating the combined translational and rotational motion pattern of exchange-bias capped Janus particles (d = 3 μm) within an artificially created magnetic field landscape in aqueous medium. The motion was studied via a light microscope with 100x magnification and a high-speed camera with 1000 frames per second.
